# The prognostic evaluation of ALBI score in endoscopic treatment of esophagogastric varices hemorrhage in liver cirrhosis

**DOI:** 10.1038/s41598-023-50629-9

**Published:** 2024-01-08

**Authors:** Yuhua Liu, Shengnan Wu, Shanshan Cai, Bushan Xie

**Affiliations:** 1 The First Affiliated Hospital, Jiangxi Medical College, Nanchang University, 17 Yongwai Street, Nanchang, 330006 Jiangxi People’s Republic of China; 2 Department of Gastroenterology, The First Affiliated Hospital, Jiangxi Medical College, Nanchang University, 17 Yongwaizheng Street, Donghu District, Nanchang, 330006 Jiangxi People’s Republic of China

**Keywords:** Liver cirrhosis, Hepatic portal vein, Stomach, Upper gastrointestinal bleeding

## Abstract

To analyze the independent risk factors for recurrent bleeding and death within 1 year after endoscopic treatment of esophagogastric varices hemorrhage (EGVB) in patients with liver cirrhosis, and to validate the predictive value of ALBI score for recurrent bleeding and death within 1 year after endoscopic treatment of EGVB in patients with liver cirrhosis. A total of 338 patients with EGVB who received endoscopic treatment for the first time in the Department of Gastroenterology, First Affiliated Hospital of Nanchang University from January 1, 2016 to March 1, 2020 were selected. A database was established to analyze the patients’ demographic data, surgical variables and postoperative outcomes. All patients were contacted and followed up to verify the predictive value of ALBI score for recurrent bleeding and mortality. 130 patients had rebleeding within 1 year after surgery (38.5%). 66 patients died within 1 year after surgery (19.5%). Patients with ALBI grade 3 had significantly higher rebleeding and mortality rates than those with grades 1 and 2. The AUC was used to compare the predictive value of the four scores for rebleeding and mortality within one year after endoscopic surgery. Both ALBI scores had the largest AUC. The ALBI score has certain predictive value for rebleeding and mortality within 1 year after endoscopic therapy in patients with cirrhotic EGVB.

## Introduction

Cirrhosis is a chronic progressive late-stage liver disease, with approximately 1.32 million deaths worldwide each year due to cirrhosis, accounting for 2.4% of total global deaths^1^. Cirrhosis is caused by various factors, such as viral infections and chronic alcoholism^2^. The liver of patients with cirrhosis undergoes diffuse fibrosis, resulting in pseudolobules and regenerative nodules. Patients with cirrhosis typically experience subtle symptoms in the compensatory phase, followed by the development of liver dysfunction and portal hypertension. Portal hypertension can lead to esophageal and gastric varices, ascites, splenomegaly, and even acute or chronic liver failure, hepatic encephalopathy, and hepatorenal syndrome^3,4^. Approximately 50% of patients with cirrhosis have esophageal and gastric varices, with an annual incidence of approximately 5–15%^5^.

Esophageal and gastric variceal bleeding is one of the most serious complications of portal hypertension in cirrhosis, accounting for 60–65% of upper gastrointestinal bleeding in cirrhosis^6,7^, with a mortality rate of up to 30% for first-time bleeding^8^. Although the prognosis of acute EGVB has improved in recent years, the mortality rate still reaches 15–20%^9^. Treatment includes fluid resuscitation, medication, and endoscopic surgery. Our country’s guidelines recommend the use of vasoactive drugs, preventive antibiotics, and proton pump inhibitors combined with endoscopic surgery to treat EGVB in patients with cirrhosis to improve patient prognosis^10^. Vasoactive drugs include terlipressin and somatostatin analogs. Endoscopic treatment includes endoscopic variceal ligation (EVL), endoscopic injection sclerotherapy (EVS), and endoscopic variceal obturation (EVO)^10^. The risk of rebleeding and mortality after successful hemostasis of EGVB in cirrhosis is high, with a rebleeding rate of up to 60% and a mortality rate of 33% within 1 year. Therefore, secondary prevention treatment is needed after successful hemostasis of EGVB for the first time, which can be achieved through drug, endoscopic, interventional, and surgical treatment measures. Currently, endoscopic treatment combined with non-selective β Nonselective beta blockers (NSBB) is the standard treatment for the secondary prevention of EGVB in cirrhosis^11^. A randomized controlled trial reported that HVPG < 12 mmHg is less likely to develop esophageal and gastric varices^12^, and HVPG > 20 mmHg is associated with failure to control bleeding in EGVB, increased risk of early rebleeding and mortality^13^. Oral NSBB can reduce HVPG by more than 20%, reducing the risk of rebleeding and mortality. Despite standardized treatment, the long-term rebleeding rate in patients with acute EGVB remains high^14,15^, and the risk of rebleeding and mortality varies greatly among different patients. Therefore, screening and early identification of high-risk patients are important, and risk stratification based on rebleeding and mortality risk is helpful for accurate assessment of prognosis and guiding treatment.

There are many prognostic models, scoring systems, and formulas that can be used for risk stratification of patients with cirrhosis and varices to predict the need for treatment, rebleeding, and mortality risk. Traditional liver function scores such as CTP score and MELD score have certain value in predicting the need for treatment, rebleeding risk, and short-term mortality of cirrhotic esophageal and gastric varices^16^, but there are certain limitations. The CTP score includes five parameters: albumin, bilirubin, PT or INR, ascites, and hepatic encephalopathy, which have problems of subjective judgment and threshold definition, and may not accurately reflect the severity of liver disease. The MELD score is mainly applicable to patients with end-stage liver disease and is not applicable to all patients with cirrhosis. Furthermore, the MELD score does not clearly define the threshold value for liver disease classification^17^.

Recently, the albumin-bilirubin score (ALBI) has been widely used in clinical practice. It is calculated using only the levels of albumin and bilirubin, and is objective, simple to obtain, easy to operate, and does not increase the economic burden of patients^18^. It was initially used to assess the liver function reserve and prognosis of patients with liver cancer^19^, and is associated with the prognosis of various chronic liver diseases. It may be more accurate than the CTP score in assessing liver function and prognosis, especially in compensated cirrhosis^20^. The ALBI score has been proven to predict the mortality risk of patients with primary biliary cirrhosis and provides better predictive performance compared to the CTP score and MELD score^21,22^. In addition, the ALBI score has also been proven to predict the adverse outcomes of patients with upper gastrointestinal bleeding due to cirrhosis^23^. However, most of the aforementioned studies have focused on the prognosis of death in patients with cirrhosis, and there are currently no studies assessing the predictive value of the ALBI score for recurrent bleeding within 1 year after endoscopic treatment in patients with cirrhosis and esophageal and gastric varices (EGVB). The aim of this study is to clarify the predictive value of the ALBI score for recurrent bleeding and mortality within 1 year after endoscopic treatment in patients with cirrhosis and EGVB and to compare its predictive performance with the CTP, MELD, and MELD-Na scoring systems. This will provide clinicians with a simple and effective non-invasive prognostic risk-scoring tool to improve the survival rate and reduce the risk of recurrent bleeding in patients with cirrhosis and EGVB.

## Results

### Patient screening

A total of 473 patients with liver cirrhosis who underwent endoscopic EGVB treatment in the Department of Gastroenterology, xx Hospital of Nanchang University from January 1, 2016, to March 1, 2020, were retrospectively screened. According to the inclusion criteria of this study, 135 patients were excluded, and 338 patients were finally included in this study.

### Comparison of basic clinical data

A total of 338 patients with EGVB in liver cirrhosis who were hospitalized in the Department of Gastroenterology, First Affiliated Hospital of Nanchang University from January 1, 2016, to March 1, 2020, were included in this study, including 257 male patients, accounting for 76%, with an average age of 52.6 ± 11.8, the oldest age of 79 years, and the youngest age of 20 years. Among them, the causes of cirrhosis were hepatitis B in 209 cases, alcohol in 35 cases, hepatitis B combined with alcohol in 18 cases, schistosomiasis in 20 cases, hepatitis C in 4 cases, biliary in 7 cases, autoimmune in 4 cases, cryptogenic in 24 cases, and other causes in 17 cases. In this study, patients were followed up for 1 year after discharge. According to the situation of rebleeding and death, patients were divided into rebleeding group and no-rebleeding group, death group, and survival group.130 EGVB patients with cirrhosis developed rebleeding within 1 year after endoscopic surgery, with a rebleeding rate of 38.5%, and 66 patients died within 1 year after surgery, with a mortality rate of 19.5%.

There were no significant differences in gender, age, length of stay, concomitant disease, cause of cirrhosis, previous EGVB, the complication of hepatic encephalopathy, bacterial infection, oral NSBB, and follow-up endoscopic therapy between rebleeding group and no-rebleeding group (P > 0.05). In the rebleeding group, 22 cases of moderate ascites (16.9%) and 18 cases of severe ascites (13.9%) were significantly higher than those in the non-bleeding group, 15 cases (7.2%) and 6 cases (2.9%), and the difference was statistically significant (P < 0.001) 0.27 cases of portal vein thrombosis in the rebleeding group (20.8%) were higher than those in the no-rebleeding group (11.1%), and the difference was statistically significant (P = 0.014). See Table 1 for details.

**Table 1:** Comparison of basic clinical data.

	No-rebleeding group	Rebleeding group	*P*	Survival group	Death group	*P*
	(n = 208)	(n = 130)		(n = 272)	(n = 66)	
Male	152 (73.1%)	105 (80.8%)	0.107	206 (75.7%)	51 (77.2%)	0.793
Age	53.0 ± 11.6	52.0 ± 12.0	0.454	52.0 ± 11.7	53.0 ± 12.3	0.438
Length of stay	10 (8–12)	10 (7–12)	0.530	10 (8–12)	10 (7–12)	0.816
Concomitant disease			0.921			0.444
Diabetes	23 (21.3%)	14 (10.8%)		30 (11.0%)	7 (11.0%)	
Hypertension	15 (7.2%)	10 (7.7%)		17 (6.1%)	8 (12.1%)	
Others	15 (7.2%)	12 (9.2%)		22 (8.1%)	5 (7.6%)	
Etiology			0.193			0.685
Hepatitis B viral	122 (58.6%)	87 (66.9%)		171 (62.9%)	38 (57.6%)	
Alcoholic	28 (13.5%)	7 (5.4%)		28 (10.3%)	7 (10.6%)	
Hepatitis B viral + alcoholic	10 (4.8%)	8 (6.2%)		13 (4.8%)	5 (7.5%)	
Schistosomiasis	10 (4.8%)	10 (7.7%)		16 (5.9%)	4 (6.1%)	
Cryptogenic	18 (8.7%)	6 (4.6%)		20 (7.4%)	4 (6.1%)	
Biliary	5 (2.4%)	2 (1.5%)		4 (1.4%)	3 (4.5%)	
Autoimmune	3 (1.4%)	1 (0.8%)		4 (1.4%)	0	
Others	12 (5.8%)	9 (6.9%)		16 (5.9%)	5 (7.6%)	
Previous EGVB	28 (13.5%)	19 (14.6%)	0.765	37 (13.6%)	10 (15.2%)	0.744
Ascites			** < 0.001**			** < 0.001**
No	135 (64.9%)	58 (44.6%)		170 (62.5%)	23 (34.8%)	
Mild	52 (25.0%)	32 (24.6%)		68 (25%)	16 (24.2%)	
Moderate	15 (7.2%)	22 (16.9%)		26 (9.6%)	11 (16.7%)	
Severe	6 (2.9%)	18 (13.9%)		8 (2.9%)	16 (24.3%)	
Hepatic encephalopathy	6 (2.9%)	4 (3.1%)	0.919	8 (2.9%)	2 (3.0%)	0.969
Bacterial infection			0.843			0.159
No	174 (83.6%)	106 (81.5%)		231 (84.9%)	49 (74.2%)	
SBP	17 (8.2%)	13 (10%)		20 (7.4%)	10 (15.2%)	
Pulmonary infection	6 (2.9%)	6 (4.7%)		8 (2.9%)	4 (6.1%)	
Intestinal infection	5 (2.4%)	2 (1.5%)		5 (1.9%)	2 (3.0%)	
Others	6 (2.9%)	3 (2.3%)		8 (2.9%)	1 (1.5%)	
Portal vein thrombosis	23 (11.1%)	27 (20.8%)	**0.014**	34 (12.5%)	16 (24.2%)	**0.016**
Oral NSBB	115 (55.3%)	62 (47.7%)	0.174	147 (54%)	30 (45.5%)	0.210
Follow-up endoscopic therapy	139 (66.8%)	90 (69.2%)	0.646	182 (66.9%)	47 (71.2%)	0.503

*SBP* spontaneous bacterial peritonitis, *Previous EGVB* previous varicose bleeding but no endoscopic treatment.

Significant values are in bold.

There were no significant differences between the death group and the survival group in gender, age, length of stay, concomitant disease, cause of cirrhosis, previous EGVB, the complication of hepatic encephalopathy, bacterial infection, oral NSBB, and endoscopic therapy (P > 0.05). The proportion of moderate ascites in the death group was 16.7% higher than that in the survival group, 9.6%; 16 cases of severe ascites in the death group (24.3%) were significantly higher than that in the survival group (2.9%), and the difference was statistically significant (P < 0.001). The proportion of portal vein thrombosis in the death group was 24.2% higher than that in the survival group (12.5%), and the difference was statistically significant (P = 0.016). See Table 1 for details.

### Comparison of laboratory indicators

There were no significant differences in WBC, PLT, NEU, TLC, ALT, Cr, BUN, Na + , and K + between the rebleeding group the and no-rebleeding group (P > 0.05). In the rebleeding group, the median Alb (28.7) was significantly lower than that in the no-rebleeding group (33.4) (P < 0.001), the median TBil (25.3) was higher than that in the non-bleeding group (18.2) (P < 0.001), and the median INR (1.37) was higher than that in the no-rebleeding group (1.23) (P < 0.001). In addition, there were statistically significant differences in RBC, Hb, AST, TC, PT, and Fib between the two groups (P < 0.05). See Table 2 for details.

**Table 2:** Comparison of laboratory indicators.

	No-rebleeding group (n = 208)	Rebleeding group (n = 130)	*P*	Survival group (n = 272)	Death group (n = 66)	*P*
RBC(× 10^12^/L)	2.82	2.55	** < 0.001**	2.82	2.51	** < 0.001**
	(2.47–3.36)	(2.19–3.05)		(2.39–3.31)	(2.19–2.84)	
Hb(g/L)	84	76	**0.002**	82	75	**0.010**
	(68–101)	(62–91)		(67–99)	(59–87)	
WBC(× 10^9^/L)	4.86	4.86	0.166	4.72	6.18	**0.006**
	(2.96–6.70)	(3.15–8.23)		(3.00–7.00)	(3.59–9.29)	
PLT(× 10^9^/L)	64	64	0.369	61	72	0.121
	(45.0–96.0)	(38.0–93.3)		(43.0–93.0)	(50.8–108.3)	
NEU(× 10^9^/L)	3.47	3.66	0.163	3.23	4.32	**0.008**
	(1.95–5.31)	(2.06–6.76)		(1.97–5.38)	(2.11–7.97)	
TLC(× 10^9^/L)	0.75	0.81	0.457	0.75	0.81	0.318
	(0.49–1.21)	(0.49–1.30)		(0.49–1.20)	(0.50–1.41)	
Alb(g/L)	33.4	28.7	** < 0.001**	32.3	27.6	** < 0.001**
	(29.7–36.9)	(25.7–32.0)		(29.0–36.5)	(23.7–30.8)	
TBil(umol/L)	18.2	25.3	** < 0.001**	19.1	28.9	** < 0.001**
	(13.3–25.5)	(17.1–36.5)		(13.6–27.6)	(18.5–40.3)	
ALT(U/L)	25	27.7	0.056	25	29	0.107
	(18.0–36.8)	(19.0–47.0)		(17.0–39.0)	(20.8–41.0)	
AST(U/L)	36	43.5	**0.011**	37	44.5	**0.016**
	(27.0–50.75)	(29.8–64.0)		(27.0–54.4)	(32.5–72.0)	
TC(mmol/L)	2.84	2.58	** < 0.001**	2.77	2.69	0.218
	(2.49–3.41)	(2.13–2.96)		(2.33–3.36)	(2.21–3.04)	
Cr(umol/L)	67.4	64	0.580	65.8	68.8	0.365
	(53.2–78.3)	(53.6–77.4)		(53.2–76.7)	(54.6–83.3)	
BUN(mmol/L)	7.01	7.76	0.120	7.12	7.55	0.361
	(4.80–9.28)	(4.65–10.80)		(4.80–9.57)	(4.45–11.40)	
Na^+^(mmol/L)	139.8	139.2	0.138	139.8	138.8	**0.003**
	(137.6–141.8)	(136.9–141.6)		(137.6–142.0)	(135.5–140.7)	
K^+^(mmol/L)	3.92	3.96	0.363	3.95	3.91	0.876
	(3.63–4.33)	(3.68–4.38)		(3.66–4.33)	(3.57–4.45)	
PT(s)	13.8	15.6	** < 0.001**	14.3	15.8	** < 0.001**
	(12.8–15.3)	(14.1–17.0)		(13.0–15.8)	(14.1–17.7)	
INR	1.23	1.37	** < 0.001**	1.26	1.38	** < 0.001**
	(1.13–1.32)	(1.26–1.50)		(1.15–1.37)	(1.24–1.57)	
Fib(g/L)	1.49	1.10	** < 0.001**	1.40	1.08	** < 0.001**
	(1.05–1.81)	(0.84–1.50)		(1.00–1.75)	(0.80–1.33)	

*NEU* neutrophils, *TLC* total lymphocyte count, *Fib* fibrinogen.

Significant values are in bold.

There were no significant differences in PLT, TLC, ALT, TC, Cr, BUN, and K + between the death group and the survival group (P > 0.05). In the death group, the median Alb (27.6) was significantly lower than that in the survival group (32.3) (P < 0.001). The median TBil (28.9) was higher than that in the survival group (19.1) (P < 0.001), and the median INR (1.38) was higher than that in the survival group (1.26) (P < 0.001).In addition, there were statistically significant differences in RBC, Hb, WBC, NEU, AST, Na + , PT, and Fib between the two groups (P < 0.05). See Table 2 for details.

### Comparison of endoscopic characteristics

There were no significant differences between the rebleeding group and the no-rebleeding group in emergency endoscopy, endoscopic site of varicose veins, type of varicose veins, varicose vein diameter, Rf, endoscopic treatment methods, endoscopic erosion, thrombosis, ulcers, and portal hypertensive gastric disease. However, the 36 cases (27.7%) that had an active hemorrhage in the rebleeding group were higher than the 32 cases (15.4%) in the non-bleeding group, and the difference was statistically significant (P = 0.006). See Table 3 for details.

**Table 3:** Comparison of endoscopic characteristics.

	No-rebleeding group (n = 208)	Rebleeding group (n = 130)	*P*	Survival group (n = 272)	Death group (n = 66)	*P*
Emergency endoscopy	103 (49.5%)	69 (53.0%)	0.524	134 (49.2%)	38 (57.6%)	0.226
Endoscopic site			0.212			0.800
Upper	131 (63.0%)	89 (68.5%)		178 (65.4%)	46 (69.7%)	
Middle	70 (33.7%)	32 (24.6%)		84 (30.9%)	18 (27.3%)	
Lower	7 (3.4%)	5 (3.8%)		10 (3.7%)	2 (3.0%)	
Type of varicose veins			0.277			0.290
Esophageal	34 (16.3%)	16 (12.3%)		42 (15.5%)	8 (12.2%)	
GOV1	131 (63.0%)	89 (68.5%)		172 (63.2%)	48 (72.7%)	
GOV1 + GOV2	5 (2.4%)	7 (5.4%)		9 (3.3%)	3 (4.5%)	
IGV	3 (1.4%)	3 (2.3%)		4 (1.5%)	2 (3.0%)	
Mixed type	35 (16.8%)	15 (11.5%)		45 (16.5%)	5 (7.6%)	
Varicose vein diameter (cm)	1.5 (1.5–1.8)	1.5 (1.2–1.8)	0.750	1.5 (1.5–1.8)	1.5 (1.2–1.8)	0.290
Endoscopic therapy			0.418			0.956
EVL	125 (60.1%)	74 (56.9%)		162 (59.5%)	37 (56.1%)	
EVO + EVS	44 (21.1%)	36 (27.7%)		63 (23.2%)	17 (25.8%)	
EVL + EVO + EVS	27 (13.0%)	16 (12.3%)		34 (12.5%)	9 (13.6%)	
Others	12 (5.8%)	4 (3.1%)		13 (4.8%)	3 (4.5%)	
Rf			0.185			0.249
0	5 (2.4%)	0		5 (1.8%)	0	
1	102 (49.0%)	62 (47.7%)		136 (50%)	28 (42.4%)	
2	101 (48.6%)	68 (52.3%)		131 (48.2%)	38 (57.6%)	
Thrombus	53 (25.5%)	42 (32.3%)	0.174	73 (26.8%)	22 (33.3%)	0.292
Erosion	42 (20.2%)	28 (21.5%)	0.766	55 (20.2%)	15 (22.7%)	0.652
Ulcer	37 (17.8%)	25 (19.2%)	0.739	48 (17.6%)	14 (21.2%)	0.502
Endoscopic active bleeding	32 (15.4%)	36 (27.7%)	**0.006**	46 (16.9%)	22 (33.3%)	**0.003**
Portal hypertensive gastropathy	154 (74.0%)	88 (67.7%)	0.208	197 (72.4%)	45 (68.2%)	0.493

*Rf* risk factor, Endoscopic active bleeding: varicose veins spurt or ooze blood under endoscopic direct vision; Emergency endoscopy: Endoscopic treatment was performed within 24 h after admission.

Significant values are in bold.

There were no significant differences between the death group and the survival group in emergency endoscopy, endoscopic site of varicose veins, type of varicose veins, varicose vein diameter, Rf, endoscopic treatment methods, endoscopic erosion, thrombosis, ulcers, and portal hypertensive gastric disease. However, the proportion of endoscopic active bleeding in the death group was 33.3% higher than that in the survival group (16.9%), and the difference was statistically significant (P = 0.003). See Table 3 for details.

### Data comparison of each scoring system

There was a significant difference in ALBI score between the rebleeding group and the no-rebleeding group (P < 0.001), and the mean ALBI score of the rebleeding group (− 1.53 ± 0.46) was significantly higher than that of the no-rebleeding group (− 2.00 ± 0.51)0.47 patients with ALBI grade 3 in the rebleeding group were higher than 21 patients in the no-rebleeding group (P < 0.001). Pairwise comparison showed that the rebleeding rate of grade 3 patients was significantly higher than that of grade 1 and grade 2 patients (P < 0.05), but there was no significant difference between the patients of grade 1 and grade 2 (P > 0.05). The median CTP score of 8 in the rebleeding group was higher than that of 6 in the no-rebleeding group (P < 0.001), and the 22 patients with CTP grade C in the rebleeding group was higher than 6 patients in the no-rebleeding group (P < 0.001). Both the median MELD score and Meld-Na score of the rebleeding group were 12, higher than those of the no-rebleeding group, which were 9 (*P* < 0.001). See Table 4 for details.

**Table 4:** Data comparison of each scoring system.

	No-rebleeding group (n = 208)	Rebleeding group (n = 130)	*P*	Survival group (n = 272)	Death group (n = 66)	*P*
ALBI score	− 2.00 ± 0.51	− 1.53 ± 0.46	< 0.001	− 1.88 ± 0.49	− 1.31 ± 0.51	< 0.001
ALBI grade			< 0.001			< 0.001
Grade 1	26 (12.5%)	4 (3.1%)		29 (10.7%)	1 (1.5%)	
Grade 2	161 (77.4%)	79 (60.8%)		212 (77.9%)	28 (42.4%)	
Grade 3	21 (10.1%)	47 (36.1%)		31 (11.4%)	37 (56.1%)	
CTP score	6 (5–7)	8 (7–9)	< 0.001	7 (6–8)	9 (7–9)	< 0.001
CTP grade			< 0.001			< 0.001
Grade A	118 (56.7%)	24 (18.5%)		133 (48.9%)	9 (13.6%)	
Grade B	84 (40.4%)	84 (64.6%)		125 (46.0%)	43 (65.2%)	
Grade C	6 (2.9%)	22 (16.9%)		14 (5.1%)	14 (21.2%)	
MELD score	9 (7–11)	12 (9–14)	< 0.001	9 (7–11)	12 (10–15)	< 0.001
MELD-Na score	9 (7–11)	12 (10–15)	< 0.001	9 (7–12)	13 (10–16)	< 0.001

There was a significant difference in ALBI score between the death group and the survival group (P < 0.001), and the mean ALBI score of the death group (− 1.31 ± 0.51) was significantly higher than that of the survival group (− 1.88 ± 0.49)0.37 patients with ALBI grade 3 in the rebleeding group were higher than 31 patients in the survival group (P < 0.001). Pairwise comparison showed that the mortality rate of grade 3 patients was significantly higher than that of grade 1 and grade 2 patients (P < 0.05), but there was no significant difference between the patients of grade 1 and grade 2 (P > 0.05). The median CTP score of 9 in the rebleeding group was higher than that of 7 in the no-rebleeding group (P < 0.001), and the proportion of patients with CTP Grade C was 21.2% in the death group, which was significantly higher than 5.1% in survival group (P < 0.001). The median MELD score of 12 and MELD-Na score of 13 in the death group were higher than those in the survival group (MELD score of 9 and Meld-NA score of 9) (P < 0.001). See Table 4 for details.

### Cox proportional hazard regression analysis of rebleeding within 1 year after endoscopic surgery

A total of 338 patients were included in this study, and 130 cases of rebleeding occurred during 1-year follow-up. Cox proportional hazard regression model was used to analyze rebleeding events. Univariate analysis showed that alcoholic cirrhosis, RBC, Hb, WBC, NEU, TLC, Alb, TBil, ALT, AST, TC, BUN, INR, Fib, ALBI score, moderate to severe ascites, portal vein thrombosis, and endoscopic active bleeding were significantly correlated with rebleeding. The above indicators related to rebleeding in univariate analysis were included in the multivariate analysis. To avoid multicollinearity, the variables contained in the ALBI score (Alb, TBil) were no longer included in the multivariate analysis. The results showed that ALBI score (HR: 3.461), INR (HR: 1.294), severe ascites (HR: 3.010), and portal vein thrombosis (HR: 2.261) were independent risk factors for rebleeding within 1 year after the endoscopy in cirrhotic EGVB patients. See Table 5 for details.

**Table 5:** COX proportional hazard regression analysis of rebleeding within 1 year after endoscopic surgery.

Influencing factor	Univariate analysis		Multivariate analysis	
	HR (95% CI)	*P*	HR (95% CI)	*P*
Gender	1.435 (0.928–2.220)	0.105		
Age	0.995 (0.980–1.009)	0.476		
Length of stay	0.981 (0.932–1.003)	0.471		
Etiology of cirrhosis				
Hepatitis B viral	1			
Alcoholic	0.433 (0.200–0.945)	0.033		
Others	0.939 (0.636–1.384)	0.749		
Concomitant disease				
No	1			
Diabetes	0.885 (0.504–1.551)	0.669		
Hypertension	1.077 (0.561–2.068)	0.823		
Others	1.303 (0.714–2.376)	0.388		
Previous EGVB	1.076 (0.661–1.751)	0.768		
Ascites				
No	1			
Mild	1.413 (0.917–2.176)	0.117	1.294 (0.834–2.009)	0.250
Moderate	2.449 (1.497–4.004)	< 0.001	1.487 (0.886–2.495)	0.133
Severe	4.721 (2.770–8.046)	< 0.001	3.010 (1.739–5.210)	** < 0.001**
Hepatic encephalopathy	1.064 (0.393–2.879)	0.903		
Bacterial infection				
No	1			
SBP	1.494 (0.839–2.659)	0.172		
Pulmonary infection	1.590 (0.698–3.620)	0.269		
Intestinal infection	0.828 (0.204–3.354)	0.791		
Others	0.841 (0.267–2.649)	0.767		
Portal vein thrombosis	2.074 (1.356–3.171)	0.001	2.261 (1.471–3.476)	** < 0.001**
Oral NSBB	0.766 (0.543–1.081)	0.129		
Follow-up endoscopic therapy	1.087 (0.749–1.577)	0.662		
RBC	0.536 (0.407–0.705)	< 0.001		
Hb	0.988 (0.981–0.996)	0.002		
WBC	1.049 (1.023–1.075)	< 0.001		
PLT	0.999 (0.995–1.002)	0.436		
NEU	1.051 (1.022–1.081)	< 0.001		
TLC	1.228 (1.020–1.478)	0.030		
Alb	0.895 (0.872–0.920)	< 0.001		
TBil	1.022 (1.014–1.030)	< 0.001		
ALT	1.001 (1.000–1.002)	0.025		
AST	1.002 (1.001–1.003)	0.002		
TC	0.620 (0.492–0.783)	< 0.001		
Cr	1.000 (0.993–1.007)	0.922		
BUN	1.019 (1.005–1.034)	0.008		
Na^+^	0.972 (0.932–1.014)	0.185		
K^+^	1.009 (0.996–1.022)	0.180		
PT	1.007 (0.994–1.019)	0.281		
INR	1.611 (1.344–1.930)	< 0.001	1.294 (1.004–1.677)	**0.046**
Fib	0.543 (0.394–0.746)	< 0.001		
ALBI score	4.321 (3.104–6.015)	< 0.001	3.461 (2.403–4.984)	** < 0.001**
Emergency endoscopy	1.167 (0.827–1.647)	0.380		
Type of varicose veins				
Esophageal	1			
GOV1	1.368 (0.803–2.330)	0.249		
Others	1.183 (0.632–2.216)	0.600		
Varicose vein diameter (cm)	0.892 (0.686–1.159)	0.392		
Endoscopic therapy				
EVL	1			
EVO + EVS	1.251 (0.840–1.864)	0.270		
EVL + EVO + EVS	1.004 (0.585–1.724)	0.987		
Others	0.581 (0.212–1.588)	0.289		
Endoscopic site				
Upper	1			
Middle	0.690 (0.461–1.031)	0.070		
Lower	0.980 (0.398–2.409)	0.964		
Endoscopic active bleeding	1.919 (1.306–2.819)	0.001		
Erosion	1.105 (0.727–1.678)	0.641		
Ulcer	1.178 (0.761–1.822)	0.463		
Thrombus	1.253 (0.867–1.810)	0.229		
Portal hypertensive gastropathy	0.772 (0.535–1.115)	0.168		

Significant values are in bold.

### Cox proportional hazard regression analysis of death within 1 year after endoscopic surgery

A total of 338 patients were included in this study, and 66 patients died during 1-year follow-up. Univariate Cox proportional hazard regression model was used to analyze death events. The results showed that RBC, Hb, WBC, NEU, TLC, Alb, TBil, ALT, AST, Na + , INR, Fib, ALBI score, moderate-severe ascites, portal vein thrombosis, bacterial infection, and endoscopic active bleeding were significantly correlated with death. The above indicators related to mortality in univariate analysis were included in multivariate analysis. To avoid multicollinearity, variables included in the ALBI score (Alb, TBil) were no longer included in multivariate analysis. Results showed that ALBI score (HR:6.991), Na + (HR:0.919), severe ascites (4.151), and portal vein thrombosis (HR:1.813) were independent risk factors for death within 1 year after endoscopic surgery in cirrhotic EGVB patients, as shown in Table 6.

**Table 6:** COX proportional hazard regression analysis of death within 1 year after endoscopic surgery.

Influencing factor	Univariate analysis		Multivariate analysis	
	HR (95% CI)	*P*	HR (95% CI)	*P*
Gender	1.145 (0.644–2.036)	0.645		
Age	1.006 (0.986–1.027)	0.545		
Length of stay	0.980 (0.912–1.053)	0.578		
Etiology of cirrhosis				
Hepatitis B viral	1			
Alcoholic	1.162 (0.519–2.613)	0.714		
Others	1.266 (0.743–2.185)	0.385		
Concomitant disease				
No	1			
Diabetes	0.997 (0.450–2.209)	0.995		
Hypertension	1.828 (0.863–3.873)	0.115		
Others	1.076 (0.427–2.707)	0.877		
Previous EGVB	1.122 (0.572–2.198)	0.738		
Ascites				
No	1			
Mild	1.714 (0.905–3.244)	0.098	1.341 (0.705–2.553)	0.371
Moderate	2.742 (1.336–5.627)	0.006	1.462 (0.693–3.084)	0.319
Severe	7.310 (3.855–13.859)	< 0.001	4.151 (2.176–7.920)	**0.002**
Hepatic encephalopathy	1.117 (0.273–4.563)	0.878		
Bacterial infection				
No	1			
SBP	2.213 (1.075–4.129)	0.030		
Pulmonary infection	2.114 (0.763–5.858)	0.150		
Intestinal infection	1.593 (0.388–6.552)	0.518		
Others	0.634 (0.088–4.592)	0.652		
Portal vein thrombosis	2.026 (1.153–3.558)	0.014	1.813 (1.028–3.197)	**0.040**
Oral NSBB	0.723 (0.445–1.174)	0.190		
Follow-up endoscopic therapy	1.176 (0.690–2.004)	0.550		
RBC	0.466 (0.312–0.696)	< 0.001		
Hb	0.984 (0.974–0.995)	0.003		
WBC	1.062 (1.035–1.089)	< 0.001		
PLT	1.002 (0.998–1.007)	0.250		
NEU	1.064 (1.033–1.095)	< 0.001		
TLC	1.375 (1.122–1.685)	0.002		
Alb	0.868 (0.836–0.901)	< 0.001		
TBil	1.028 (1.018–1.038)	< 0.001		
ALT	1.001 (1.000–1.002)	0.040		
AST	1.002 (1.000–1.003)	0.021		
TC	0.821 (0.596–1.130)	0.226		
Cr	1.007 (0.099–1.105)	0.099		
BUN	1.022 (0.099–1.045)	0.066		
Na^+^	0.924 (0.876–0.974)	0.003	0.919 (0.870–0.970)	**0.002**
K^+^	0.970 (0.848–1.110)	0.659		
PT	1.011 (0.996–1.026)	0.140		
INR	1.433 (1.102–1.865)	0.007		
Fib	0.494 (0.312–0.781)	0.003		
ALBI score	7.352 (4.509–11.989)	< 0.001	6.991 (4.041–12.095)	** < 0.001**
Emergency endoscopy	1.334 (0.825–2.190)	0.235		
Type of varicose veins				
Esophageal	1			
GOV1	1.455 (0.688–3.076)	0.326		
Others	0.917 (0.362–2.325)	0.856		
Varicose vein diameter (cm)	0.797 (0.531–1.197)	0.274		
Endoscopic therapy				
EVL	1			
EVO + EVS	1.116 (0.628–1.981)	0.780		
EVL + EVO + EVS	1.126 (0.543–2.333)	0.750		
Others	0.943 (0.291–3.058)	0.922		
Endoscopic site				
Upper	1			
Middle	0.824 (0.478–1.422)	0.487		
Lower	0.791 (0.192–3.257)	0.745		
Endoscopic active bleeding	2.253 (1.350–3.759)	0.002		
Erosion	1.246 (0.690–2.248)	0.466		
Ulcer	1.143 (0.643–2.033)	0.649		
Thrombus	1.284 (0.770–2.143)	0.338		
Portal hypertensive gastropathy	0.792 (0.472–1.330)	0.378		

Significant values are in bold.

### Survival curve analysis of cumulative rebleeding rate in patients with different ALBI grades

**Figure 1:**
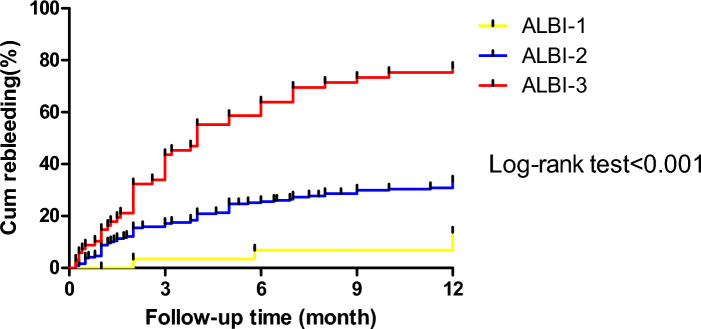
Cumulative rebleeding rates of patients with different ALBI grades.

The median time without bleeding in all patients was 8.8 months (95% CI 8.3–9.3). The median time without bleeding was 11.4 months (95% CI 10.6–12.3) in ALBI Grade 1 patients, 9.3 months (95% CI 8.8–9.9) in ALBI grade 2 patients, and 5.5 months (95% CI 4.5–6.6) in Albi grade 3 patients. The cumulative rebleeding rate of all patients was 38.5% (130 cases). The cumulative rebleeding rate of 69.1% in grade 3 patients was significantly higher than that in grade 1 patients (13.3% (χ^2^ = 30.034, P < 0.001) and grade 2 patients (32.9% (χ^2^ = 45.143, P < 0.001). See Fig. 1 for details.

### Survival curve analysis of cumulative mortality rate in patients with different ALBI grades

**Figure 2:**
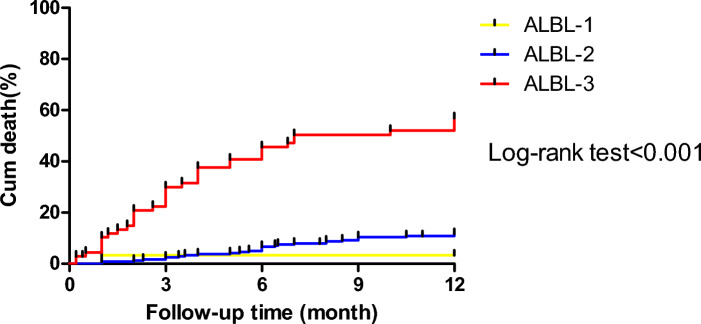
Cumulative mortality of patients with different ALBI grades.

The median survival time of all patients was 10.6 months (95% CI 10.2–10.9), the median survival time was 11.6 months (95% CI 10.9–12.3) of ALBI grade 1 patients, 11.3 months (95% CI 11.0–11.5) of ALBI grade 2 patients, and 7.5 months (95% CI 6.4–8.7) for ALBI grade 3 patients. The cumulative mortality rate for all patients was 19.5%. The cumulative mortality rate of patients with ALBI grade 3 was 54.4%, which was significantly higher than that of ALBI grade 1 patients, which was 3.3%(χ^2^ = 21.333, P < 0.001) and ALBI grade 2 patients, which was 11.7%(χ^2^ = 81.833, P < 0.001). See Fig. 2 for details.

### Correlation analysis of ALBI score and other scores

Spearman correlation analysis was used to evaluate the correlation between ALBI score and CTP score, MELD score, and MELD-Na score, and it was found that ALBI score was significantly positively correlated with CTP score, MELD score and Meld-NA score (r values were 0.781, 0.513, 0.466, respectively). P values < 0.001), see Table 7 for details.

**Table 7:** Correlation analysis between ALBI score and each score.

	ALBI score	
	Correlation coefficient r	*P*
CTP score	0.781	< 0.001
MELD score	0.513	< 0.001
MELD-Na score	0.466	< 0.001

### Comparison of ALBI score, CTP score, MELD score, and Meld-Na score in predicting rebleeding within 1 year after endoscopic surgery

**Figure 3:**
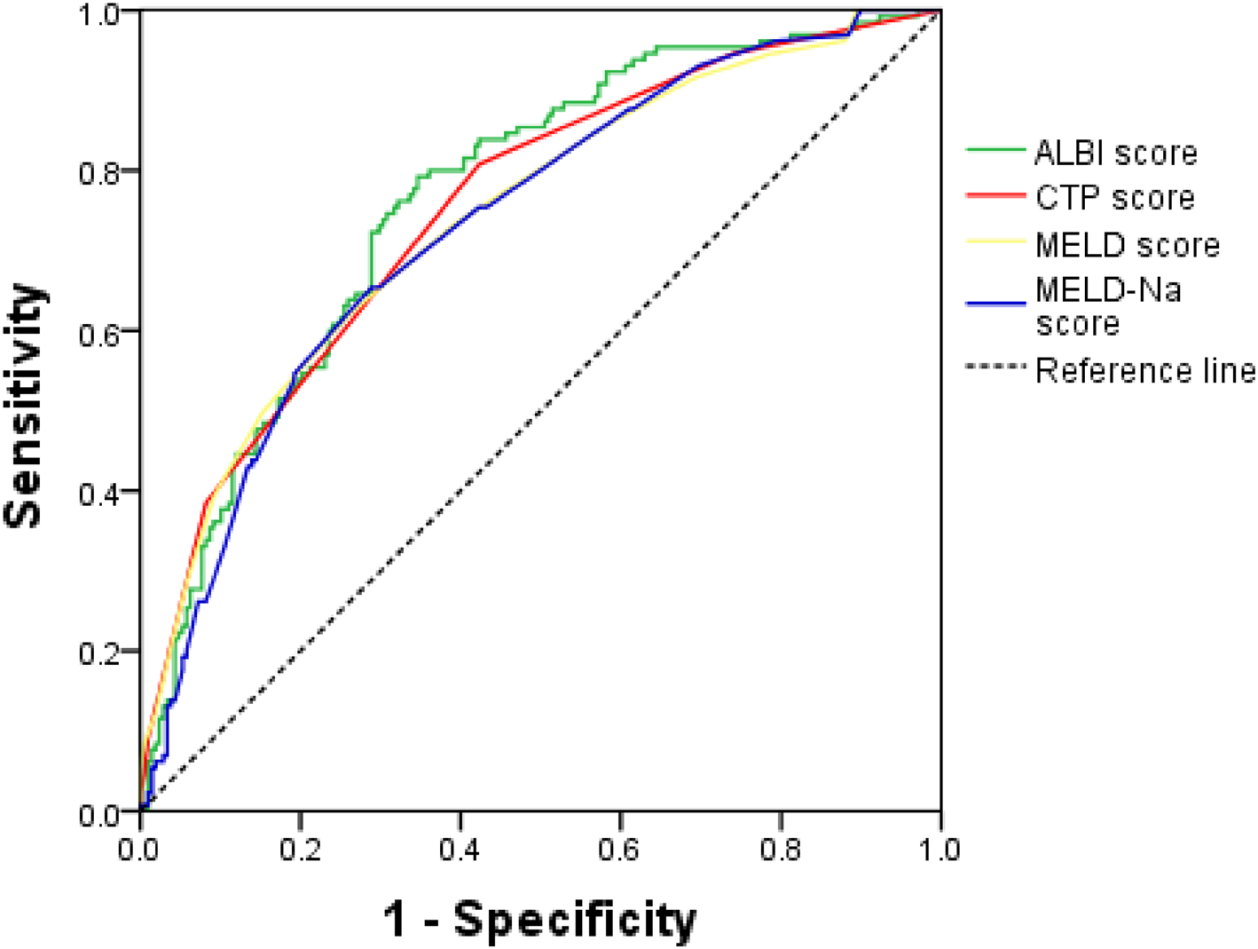
ROC curve of four scores predicting rebleeding.

ROC curve was used to evaluate the predictive value of four scores for rebleeding within 1 year after endoscopic surgery. The results showed that the AUC of ALBI score, CTP score, MELD score, and Meld-NA score were 0.765, 0.752, 0.743, and 0.733, respectively, among which the AUC of ALBI score was the largest, followed by CTP score, and MELD-Na was the smallest (Table 8, Fig. 3). There was no statistical difference in the predictive efficacy of the four scores in determining the rebleeding rate of patients within 1 year (all P values > 0.05).

**Table 8:** ROC curve of four scores for predicting rebleeding.

Prognostic score	AUC (95% CI)	*P*	Cut-off value	Sensitivity (%)	Specificity (%)	Youden’s index
ALBI	0.765 (0.716–0.809)	< 0.001	− 1.8	79.2	64.9	0.44
CTP	0.752 (0.703–0.798)	< 0.001	6	80.8	57.7	0.38
MELD	0.743 (0.693–0.788)	< 0.001	10	62.3	73.6	0.36
MELD-Na	0.733 (0.683–0.780)	< 0.001	10.7	65.4	71.2	0.37
ALBI vs CTP	0.012 (− 0.029–0.053)	0.556				
ALBI vs MELD	0.022 (− 0.037–0.081)	0.463				
ALBI vs MELD-Na	0.031 (− 0.031–0.094)	0.463				
CTP vs MELD	0.010 (− 0.048–0.068)	0.741				
CTP vs MELD-Na	0.019 (− 0.042–0.080)	0.537				
MELD vs MELD-Na	0.009 (− 0.018–0.036)	0.496				

*Cut-off value* optimal cut-off value, *AUC* area under the curve.

### Comparison of ALBI score, CTP score, MELD score, and Meld-Na score in predicting death within 1 year after endoscopic surgery

**Figure 4:**
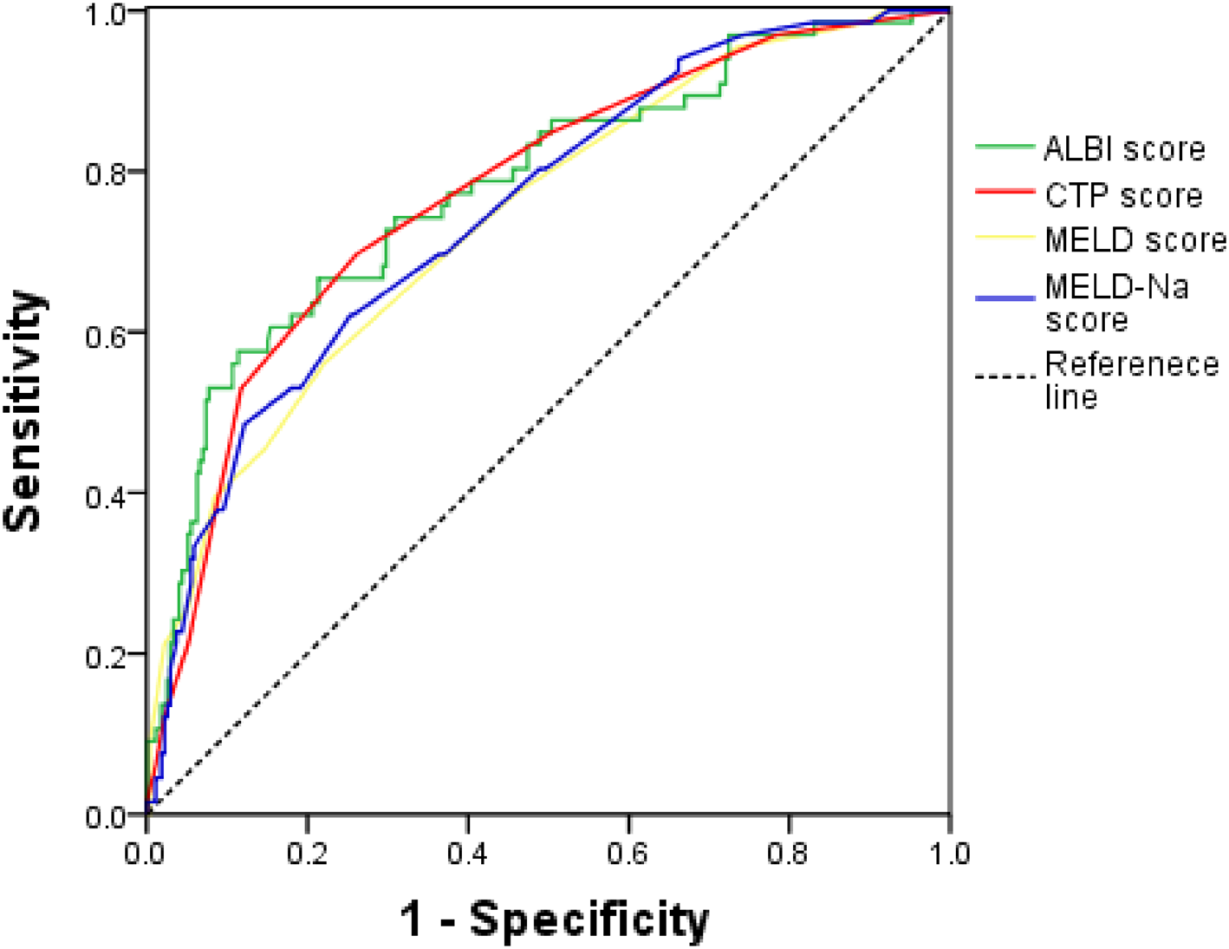
ROC curve of four scores predicting death.

ROC curve was used to evaluate the predictive value of four scores for the death of patients within 1 year after endoscopic surgery. The results showed that the AUC of ALBI score, CTP score, MELD score, and Meld-Na score were 0.780, 0.774, 0.741, and 0.751, respectively, among which the AUC of ALBI score was the largest, followed by CTP score, and MELD score was the smallest (Table 9, Fig. 4). Compared with each score, there was no statistical difference in the predictive efficacy of the four scores in determining the mortality of patients within 1 year (all P values > 0.05).

**Table 9:** ROC curve of four scores for predicting death.

Prognostic score	AUC (95% CI)	*P*	Cut-off value	Sensitivity (%)	Specificity (%)	Youden’s index
ALBI	0.780 (0.732–0.823)	< 0.001	− 1.41	57.6	87.9	0.45
CTP	0.774 (0.726–0.818)	< 0.001	7	69.7	73.9	0.44
MELD	0.741 (0.691–0.787)	< 0.001	11	56.1	77.9	0.34
MELD-Na	0.751 (0.701–0.796)	< 0.001	11.5	62.1	74.6	0.37
ALBI vs CTP	0.006 (− 0.041–0.052)	0.812				
ALBI vs MELD	0.038 (− 0.035–0.112)	0.305				
ALBI vs MELD-Na	0.029 (− 0.053–0.110)	0.492				
CTP vs MELD	0.033 (− 0.034–0.099)	0.333				
CTP vs MELD-Na	0.023 (− 0.048–0.094)	0.526				
MELD vs MELD-Na	0.010 (− 0.028–0.048)	0.614				

*Cut-off value* optimal cut-off value, *AUC* area under the curve.

## Discussion

Acute EGVB is a serious complication in patients with cirrhosis and portal hypertension. Although the prognosis has improved in recent years, the mortality rate remains high at 15–20%, with a high rate of recurrent bleeding^24^. It has been reported that the early recurrent bleeding rate is about 30% to 40% within the first 6 weeks after the first occurrence of EGVB in patients with cirrhosis, and the recurrent bleeding rate within 1 year reaches 60%^25^. Therefore, timely and accurate prognosis assessment, as well as the use of prediction models to screen high-risk patients for personalized comprehensive prevention and treatment to improve prognosis, are hot topics in current research. Commonly used CTP and MELD scores can assess prognosis, but there are certain limitations. The ALBI score can predict adverse outcomes, but there are relatively few studies on its prediction of EGVB recurrent bleeding. This study aims to analyze the independent risk factors for recurrent bleeding and mortality, validate the predictive value of ALBI for recurrent bleeding and mortality in EGVB, and compare its predictive performance with CTP, MELD, and MELD-Na scoring systems.

This study included 338 patients with cirrhosis and acute EGVB. After 1 year of follow-up, the recurrent bleeding rate was 38.5%, and the mortality rate was 19.5%. A study by Hu et al.^26^ reported a recurrent bleeding rate of 40% within 1 year after endoscopic treatment in patients with cirrhosis and EGVB, which is similar to the recurrent bleeding rate of 38.5% in this study. In a retrospective cohort study by Salman et al.^27^, the recurrent bleeding rate within 1 year after endoscopic treatment for acute variceal hemorrhage was 28%, which is lower than that in this study. It is considered that this study included only half of the cases included in this study, and the subjects were limited to esophageal varices, while the subjects in this study included gastric varices. Previous literature suggests that patients with gastric variceal bleeding have a significantly higher recurrent bleeding rate after surgery than patients with esophageal varices^28^. Lian et al.^29^ showed in an analysis of factors affecting recurrent bleeding after endoscopic treatment in patients with cirrhosis and acute EGVB from 2017 to 2018 that the recurrent bleeding rate within 1 year was 44.6%, which is higher than that in this study. It is considered that this may be related to the inclusion of patients with liver cancer, and this study excluded patients with liver cancer. It has been reported that cirrhosis patients with liver cancer have a poor prognosis and an increased risk of recurrent bleeding^23^. Most previous studies^5^ have shown that the mortality rate after endoscopic treatment for acute EGVB is about 20%. This study showed that the mortality rate within 1 year after endoscopic surgery in patients with cirrhosis and EGVB was 19.5%, which is basically consistent with previous studies. Xavier et al.^17^ found in a retrospective study of acute upper gastrointestinal bleeding in cirrhosis that the mortality rate within 1 year was 19.8%, which is almost the same as this study, although the patients included in this study were not limited to acute EGVB patients, but the majority of the causes of bleeding were variceal hemorrhages (75.5%). Cho et al.^14^ revealed in a retrospective study that the mortality rate within 1 year after endoscopic surgery in patients with EGVB was 26%, which is higher than the mortality rate of 19.5% in this study. It is considered that this study included a high proportion of liver cancer patients (35.1%) and CTP grade C patients (40.1%), while this study excluded liver cancer patients and CTP grade C patients accounted for less than 10%. Patients with liver cancer and CTP grade C have generally poor liver function and high mortality risk.

This study used the Cox proportional hazards regression model to analyze the factors affecting recurrent bleeding within 1 year after endoscopic surgery in patients with cirrhosis and EGVB. It was found that ALBI score, INR, severe ascites, and portal vein thrombosis were independent risk factors. The ALBI score, originally used to assess the prognosis of liver cancer patients, includes two indicators: albumin and total bilirubin. It can also be used to evaluate the prognosis of patients with cirrhosis and acute EGVB^30,31^. Albumin has multiple functions, including volume expansion, antioxidant, immunomodulation, and anti-inflammatory effects, which can be used for the treatment of decompensated cirrhosis. Some studies^32^ have shown that bacterial infection may increase portal pressure and risk of recurrent bleeding, while albumin may potentially prevent recurrent bleeding by regulating hemostasis, vessel relaxation, and acid–base balance. Hypoalbuminemia is considered an indicator of the severity of liver dysfunction^33^. Wang et al.^34^ found that albumin infusion can reduce the risk of recurrent bleeding and hospital death in patients with cirrhosis and EGVB. Univariate analysis in this study suggested that albumin was a protective factor for recurrent bleeding (0.895). Similarly, other studies^26^ have shown a significant correlation between high bilirubin levels and recurrent bleeding rates, and the ALBI score includes these two indicators, strongly suggesting its effectiveness in predicting recurrent bleeding risk. It has been reported^23^ that the ALBI score can predict adverse outcomes in acute upper gastrointestinal bleeding in cirrhosis, including recurrent bleeding and death. This study found that the ALBI score was an independent risk factor for recurrent bleeding within 1 year after surgery in patients with cirrhosis and acute EGVB. In addition, previous studies have shown a direct correlation between HVPG and recurrent bleeding in cirrhosis EGVB^35^, and some studies have reported a good correlation between the ALBI score and HVPG. Among non-invasive indicators, the ALBI score has the best correlation with HVPG and can be used as a predictor of recurrent bleeding in cirrhosis patients^36^.

Duenas et al.^37^ noted that an elevated INR is associated with recurrent bleeding after ligation of EGVB in cirrhotic patients. Faisal et al.^38^ found that an increased INR value can accurately predict early recurrent bleeding after ligation. Zhang et al.^39^ used a Cox proportional hazards regression model in a study on the correlation between probiotics and recurrent bleeding of EGVB in cirrhosis, and found that the INR was an independent risk factor for recurrent bleeding within 1 year (HR: 1.697). Our study also concluded that the INR is an independent risk factor for recurrent bleeding within 1 year (HR: 1.294). The INR has been proven to be an indicator reflecting the coagulation status of cirrhotic patients. An elevated INR suggests decreased liver synthetic function and reduced coagulation factor synthesis. This may explain the association between an elevated INR and recurrent bleeding.

Studies^40–42^ have shown that ascites are a risk factor for rebleeding after endoscopic treatment of acute EGVB in patients with cirrhosis. This study shows that severe ascites are an independent risk factor for rebleeding within one year after endoscopic treatment in patients with EGVB. Considering that ascites are common in patients with decompensated cirrhosis, patients with severe ascites have a worse liver function status than those without ascites, often in a hyperdynamic circulatory state, with higher portal pressure, resulting in unstable hemodynamics, reduced hepatic rebleeding, poorer vascular condition and high tension in varicose veins, which may be more prone to bleeding.

Gao et al.^43^ found in a retrospective analysis that portal vein thrombosis is associated with recurrent bleeding after acute EGVB endoscopic treatment. The recurrent bleeding rate at 6 weeks with portal vein thrombosis (11.92%) was significantly higher than without portal vein thrombosis (1.83%). Multivariate analysis showed that portal vein thrombosis is an independent risk factor for recurrent bleeding at 6 weeks after endoscopic treatment. Huang Xiaozhuan et al.^44^ believed that portal vein thrombosis formation is an independent risk factor for recurrent bleeding within 1 year after endoscopic treatment of hepatitis B cirrhosis EGVB, which is basically consistent with the conclusion of this study. Portal vein thrombosis is a common complication of cirrhosis, characterized by thrombus formation in the portal vein, involving both the left and right branches, which can extend to the superior mesenteric vein and splenic vein. Portal vein thrombosis can lead to increased portal pressure, reduced blood flow to the liver, causing intestinal congestion and edema, bacterial translocation, and liver dysfunction, thereby increasing the risk of recurrent bleeding.

In addition, univariate analysis found that endoscopic active bleeding was associated with rebleeding, but multivariate analysis showed that the difference did not reach statistical significance. However, previous studies^45^ have suggested that patients with endoscopic active bleeding have a higher risk of rebleeding compared to those without endoscopic active bleeding, suggesting that such patients should be actively followed up with endoscopic treatment or oral NSBB and other secondary prevention measures to reduce the risk of rebleeding.

The results of multivariate Cox proportional hazards regression model analysis showed that ALBI score, Na + , severe ascites, and portal vein thrombosis were independent risk factors for death within 1 year after endoscopic treatment in patients with cirrhosis EGVB. Some studies^46,47^ have suggested that the ALBI score can accurately predict the severity of illness and long-term prognosis in patients with post-hepatitis B cirrhosis, and is an independent risk factor for death. The ALBI score is a predictor of cirrhosis mortality, including two parameters of albumin and bilirubin. In the decompensated stage of cirrhosis, low albumin and high bilirubin levels indicate severe liver dysfunction and poor prognosis. Sun Mengyuan^30^ and Li Dezhao et al.^31^ separately conducted retrospective studies on the prognosis of patients with cirrhosis EGVB within 1 year after endoscopic surgery, and both studies showed that the ALBI score was an independent risk factor for death, which is consistent with the results of this study, but with a larger sample size.

Zou et al.^48^ studied the prediction of low serum sodium as a factor reducing survival rates in patients with cirrhosis. They found that cirrhotic patients with hyponatremia were mainly due to free water retention and were positively correlated with the severity of portal hypertension or HVPG^49^. This study showed that Na + was an independent risk factor for death within 1 year after endoscopic surgery in patients with cirrhosis EGVB. Although this study only focused on patients with cirrhosis EGVB, EGVB is a common complication of cirrhosis, so low serum sodium can still be considered a predictor of survival in patients with cirrhosis EGVB.

Some studies^41^ have suggested that ascites is an independent risk factor for survival in patients with cirrhosis EGVB. This study found that severe ascites was an independent risk factor for death within 1 year after endoscopic surgery in patients with cirrhosis EGVB, which may be due to the high incidence of hyponatremia, spontaneous bacterial peritonitis, hepatorenal syndrome, hepatic encephalopathy, and other complications in patients with severe ascites. This suggests a poor prognosis and increased risk of death. The study also found that severe ascites was an independent risk factor for recurrent bleeding, which also led to a poor prognosis and increased risk of death.

In addition, multivariate analysis showed that portal vein thrombosis was an independent risk factor for death after acute EGVB endoscopic surgery. Xiao et al.^42^ also found that portal vein thrombosis was an independent risk factor for long-term mortality after endoscopic surgery in patients with cirrhotic portal hypertension. Patients with portal vein thrombosis had a significantly higher mortality rate than those without, possibly due to the increase in portal pressure caused by portal vein thrombosis, which increased the risk of ascites and variceal bleeding, leading to an increased risk of death.

This study compared the predictive performance of ALBI score, CTP score, MELD score, and MELD-Na score for predicting risk of recurrent bleeding and death within 1 year in patients with cirrhotic portal hypertension by drawing ROC curves. The results showed that for predicting risk of recurrent bleeding, the AUCs of ALBI score, CTP score, MELD score, and MELD-Na score were 0.765, 0.752, 0.743, and 0.733, respectively. Among them, the ALBI score had the largest AUC, but there was no significant difference in predictive performance between each score (P values were all > 0.05). For predicting risk of death, the AUCs of ALBI score, CTP score, MELD score, and MELD-Na score were 0.780, 0.774, 0.741, and 0.751, respectively. The ALBI score had the largest AUC, but there was no significant difference in predictive performance between each score (P values were all > 0.05).

This study is the first to find that the ALBI score has moderate predictive accuracy for assessing the risk of recurrent bleeding within 1 year after surgery in patients with cirrhotic portal hypertension. In addition, correlation analysis of the four scores showed that the ALBI score had the strongest correlation with the CTP score (R value of 0.781), slightly weaker correlation with the MELD and MELD-Na scores (R values of 0.513 and 0.466, respectively). It is generally believed that the MELD and MELD-Na scores are mainly applicable to patients with advanced cirrhosis, so for predicting recurrent bleeding, the ALBI score and CTP score may be more valuable. Some studies^47^ have suggested that in patients with hepatitis B-related cirrhosis, prognosis may be different for patients with the same CTP grade but different ALBI grades. In this study, for predicting recurrent bleeding, the ALBI score showed similar or better predictive performance compared to the CTP score. Furthermore, as an objective and effective scoring system with only two parameters that is easy to obtain objectively and convenient to use, the ALBI score may be able to replace the CTP score to identify high-risk patients for recurrent bleeding.

The ALBI score also has moderate predictive accuracy for assessing the risk of death within 1 year after surgery in patients with cirrhotic portal hypertension. Li Dezhao et al.^31^ studied the predictive performance of the ALBI score in combination with CTP and MELD-Na scores for predicting mortality risk in patients with cirrhotic portal hypertension one year after endoscopic surgery, which is consistent with this study. Xavier et al.^17^ also reported in their study that the ALBI score accurately predicted mortality during hospitalization and at 30 days, while CTP and MELD scores did not predict these outcomes. However, for predicting one-year mortality, all three scores showed similar predictive performance. This study found that the four scores had similar predictive performance for patient mortality within one year after surgery. The CTP score remained effective for assessing mortality risk in patients with cirrhotic portal hypertension, which is consistent with the report by Zhao et al.^50^.

Patients with cirrhotic portal hypertension (EGVB) undergoing endoscopic surgery have a high risk of recurrent bleeding and death. It is crucial to rapidly and accurately risk-stratify these patients. In this study, the ALBI score was an independent risk factor for recurrent bleeding and death within 1 year after endoscopic surgery in patients with cirrhotic EGVB. Although the predictive performance of the ALBI score was similar to that of the CTP score, MELD score, and MELD-Na score in this study, it had a limited sample size and was retrospective in nature, which may have introduced selection bias. In addition, the study did not consider factors such as the severity of esophageal varices, portal vein size, antiviral treatment, differences in secondary prevention after surgery, and follow-up outcomes obtained through family member, which may have introduced recall bias. Finally, the study population was limited to patients with acute EGVB. Therefore, the conclusions of this study are subject to some limitations and require validation in multi-center, large-sample, and prospective studies.

## Method

### Subjects

Patients with EGVB in liver cirrhosis who underwent endoscopic treatment for the first time in the Department of Gastroenterology, First Affiliated Hospital of Nanchang University from January 1, 2016, to March 1, 2020, were selected.

### Inclusion criteria

(1) Patients were diagnosed with cirrhosis; (2) Within 5 days before admission (120 h), patients had melaena or/and hematemesis, fecal occult blood test positive and other signs of upper gastrointestinal hemorrhage, esophagogastric varicose jet bleeding, blood oozing, red thrombus, white thrombus, or simple esophagogastric varices under gastroscopy, and endoscopic treatment was performed at the same time.

### Exclusion criteria

(1) Patients who had previously received endoscopic treatment for acute EGVB; (2) Patients with upper gastrointestinal hemorrhage in the non-acute phase (5 days); (3) Patients with concurrent liver cancer or other malignant tumors; (4) Patients with severe primary cardiac, pulmonary, or renal insufficiency; (5) Patients under 18 years of age or over 80 years of age; (6) Patients who refuse endoscopic therapy; (7) Patients whose phone information is missing or who refuse follow-up visits; (8) Patients who underwent liver transplantation, TIPS, surgical hemostasis, splenectomy, or splenic embolization before; (9) Patients who underwent liver transplantation, TIPS, surgical hemostasis, splenectomy, or splenic embolization in the follow-up period; (10) Patients with incomplete case data.

### Treatment

All liver cirrhosis patients with EGVB fasted immediately after admission and were treated with blood volume supplement, vasoactive drugs (including terlipressin, somatostatin, or similar) to reduce portal pressure, proton pump inhibitors to suppress acid, prophylactic antibiotics, and other medical drugs. Endoscopic examination and treatment were performed after hemodynamic stability and contraindication was excluded (within 48 h after admission). After the operation, the bleeding situation of the patients was observed, fasting and water prohibition were continued, proton pump inhibitor therapy, somatostatin, or similar were given to reduce portal pressure, and terlipressin was combined when necessary.

### Collect indexes

Data of patients were collected through hospital review, medical records, and telephone consultation, which include: 1. Basic clinical data (general information (gender, age, length of stay, cause of cirrhosis, concomitant diseases, and previous history of EGVB), clinical symptoms and complications (ascites, bacterial infection, hepatic encephalopathy, portal vein thrombosis, etc.), whether long-term oral NSBB and endoscopic treatment were followed up after discharge); 2. Results of the first laboratory examination after admission (WBC, RBC, Hb, PLT, NEU, TLC, Alb, TBil, AST, ALT, Cr, BUN, TC, Na + , K + , PT, INR, and Fib); 3. Endoscopic characteristics (varicose vein type, varicose vein endoscopic site, varicose vein diameter, endoscopic treatment method, Rf, presence of varicose veins (active bleeding, erosion, ulcers, or thrombosis), emergency endoscopic therapy, portal hypertensive gastropathy, etc.); 4. Data of each scoring system (ALBI score, CTP score, MELD score, and Meld-Na score).

### Follow-up contents

In this study, the follow-up period was from admission to 1 year after endoscopic therapy. Follow-up was carried out through hospitalization, medical records, and telephone calls. The primary endpoint of follow-up was rebleeding and the secondary endpoint of follow-up was death. It was terminated when the death occurred or the follow-up was for 1 year. Follow-up included whether and time of rebleeding, and whether and time of death. According to the outcome, the patients were divided into rebleeding group, no-rebleeding group, death group, and survival group.

### ALBI score and various score calculation methods

The calculation methods of ALBI score, CTP score, MELD score, and MELD-Na score are shown in Table 10.

**Table 10:** Calculation method of each score.

Prognostic score	Formula		
ALBI	− 0.085 × [Alb(g/L)] + 0.66 × lg[TBil(umol/L)]; grade 1 ≤ -2.6, -2.6 < 2 grade ≤ -1.39, grade 3 > -1.39。		
MELD	3.78 × ln[TBil(umol/L)] + 11.2 × ln(INR) + 9.57 × ln[Cr(umol/L)] + 6.4 × [etiology (0 for biliary or alcoholic cirrhosis, 1 for other causes)]		
MELD-Na	MELD + 1.59 × [135-Na^+^ (mmol/L)], Na^+^ > 135 mmol/L, calculated as 135 mmol/L, Na^+^ < 120 mmol/L, calculated as 120 mmol/L		
CTP score			
Clinical biochemical index\score	1	2	3
Hepatic encephalopathy (Grade)	No	I–II	III–IV
Ascites	No	Mild	Moderate/severe
TBil (umol/L)	< 34	34–51	> 51
Albumin (g/L)	> 35	28–35	< 28
PT/INR prolong, second	< 4	4–6	> 6

Grade A: 5–6 scores, Grade B: 7–9 scores, Grade C: ≥ 10 scores.

### Statistical methods

SPSS25.0 and MedCalc were used for statistical analysis. GraphPad Prism9 was used to draw the statistical graphs. Normal distributed continuous variables were represented by mean + standard deviation (SD) and compared by independent *t* test. Continuous variables with non-normal distribution were represented by median (25th percentile-75th percentile) and compared by the Mann–Whitney *U* test. Classification variables were expressed as frequency and proportion, which were compared by the chi-square test or Fisher’s exact test. Spearman correlation analysis was used to evaluate the correlation between the two scoring systems. This study uses backward elimination to select predicting variables in a multivariate analysis. Univariate and multivariate Cox proportional hazard regression models were used to analyze the relationship between the relevant factors and the prognosis (rebleeding and death). Cumulative rebleeding rate and cumulative mortality were estimated by Kaplan–Meier analysis and compared by log-rank test. ROC curve was drawn and AUC was calculated. The AUC of each scoring system was compared by DeLong test. P < 0.05 was statistically significant.

#### Statements

The authors declare that all methods were carried out in accordance with relevant guidelines and regulations.

All experimental protocols were approved by the First Affiliated Hospital of Nanchang University.

Informed consent was obtained from all subjects and/or their legal guardian(s).

## Data Availability

The following information was supplied regarding data availability: The raw data and codebook are available in the Supplemental Files.
